# Auditory complications of COVID-19 infection and vaccination: a TriNetX retrospective cohort study

**DOI:** 10.1007/s00405-026-10512-5

**Published:** 2026-07-27

**Authors:** Warren B. Chun, Kaiwen Chen, Shaun A. Nguyen, Robert F. Labadie

**Affiliations:** 1 Department of Otolaryngology– Head & Neck Surgery, Medical University of South Carolina, 135 Rutledge Ave, MSC 550, Charleston, SC 29425 USA; 2 Rutgers Robert Wood Johnson Medical School, New Brunswick, NJ USA

**Keywords:** COVID-19, Vaccination, Tinnitus, Hearing loss, TriNetX

## Abstract

**Purpose:**

To assess the incidence of new-onset tinnitus and hearing loss following COVID-19 infection or vaccination using a large, real-world electronic health record database.

**Methods:**

A retrospective cohort study utilizing the TriNetX Global Collaborative Network (180 + million records). Adults without prior tinnitus or hearing loss were grouped by exposure: COVID-19 infection, mRNA vaccination, viral vector vaccination, and unexposed controls. Outcomes included tinnitus (ICD-10-CM: H93.1) and hearing loss (ICD-10-CM: H90) within six months post-index. Propensity score matching controlled for confounding and odds ratios (ORs) were calculated.

**Results:**

mRNA vaccination was associated with increased odds of tinnitus (OR 1.220; 95% CI: 1.189–1.252) and hearing loss (OR 1.214; 95% CI: 1.189–1.240), with small absolute risk differences (0.143% and 0.215%, respectively; *p* < 0.0001). Viral vector vaccination showed increased tinnitus odds (OR 1.269; 95% CI: 1.114–1.447) but not hearing loss. COVID-19 infection was not associated with tinnitus (OR 1.022; 95% CI: 0.997–1.048) and showed modestly reduced odds of hearing loss (OR 0.930; 95% CI: 0.911–0.950) versus controls. Compared directly, infection conferred lower tinnitus odds and higher hearing loss odds than mRNA vaccination ([OR 0.856; 95% CI: 0.833–0.879] and [OR 1.313; 95% CI: 1.285–1.343], respectively).

**Conclusion:**

COVID-19 infection was not associated with tinnitus or hearing loss. mRNA and viral vector vaccines showed small but significant increases in new-onset tinnitus, with mRNA vaccines also associated with hearing loss. While absolute risks remained low, findings support continued surveillance and research to inform counseling and promote understanding of potential otologic effects.

## Introduction

Severe acute respiratory syndrome coronavirus 2 (SARS-CoV-2), the causative agent of coronavirus disease 2019 (COVID-19), remains a global health challenge with far-reaching clinical manifestations extending beyond the respiratory system. Emerging evidence indicates that auditory symptoms, including tinnitus and hearing loss, may be associated with COVID-19 infection, and there is growing concern regarding similar outcomes following vaccination [1–4]. While hearing loss is a well-recognized sequela of viral infections, SARS-CoV-2 appears to impact the auditory apparatus by mechanisms that could include direct viral invasion, immune-mediated responses, and systemic inflammation, thereby affecting cochlear and neural function [5–7].

The prevalence of auditory symptoms in adults post-COVID-19 infection is notable: meta-analyses estimate the incidence of hearing loss at approximately 8.2% and tinnitus at about 14%, though most nonhospitalized patients exhibit symptom improvement over time [8–9]. Sudden sensorineural hearing loss has been documented across age groups, including young adults, with data suggesting that COVID-19 infection may independently elevate risk for both conductive and sensorineural hearing loss [10–14]. Persistent neurotologic sequelae, such as tinnitus, have also been observed as part of post-acute sequelae of SARS-CoV-2 (PASC) or “long COVID,” potentially linked to central auditory pathway alterations and ongoing immune activity [2, 9, 15–17].

The rapid development and deployment of COVID-19 vaccines, including mRNA platforms (e.g. Pfizer-BioNTech, Moderna) and viral vector vaccines (e.g. Janssen), has been instrumental in curbing the pandemic. However, vaccine-related auditory side effects, particularly tinnitus and hearing loss, have also been reported. Data from passive surveillance systems suggest that tinnitus may be more likely to develop or worsen following vaccination, with the highest likelihood following the Janssen vaccine [3, 18]. While some studies note increased incidence of tinnitus after all vaccine types, the magnitude of risk remains low overall, and most impacted individuals experience improvement within weeks to months [3, 18–4]. No substantial difference in onset latency or severity has been noted between mRNA and non-mRNA vaccine platforms, suggesting etiological complexity beyond vaccine formulation alone [3, 18].

Despite the recognition of these symptoms, the precise epidemiology, pathophysiology, and prognosis of COVID-19- and vaccine-associated auditory dysfunction remain incompletely understood. Large-scale, real-world analyses are needed to delineate the extent to which these exposures contribute to the onset of tinnitus and hearing loss relative to background rates in the general population. A prior TriNetX study demonstrated that new-onset tinnitus within 21 days of COVID-19 vaccination was rare and less common than other vaccinations [19]. In our study, we leverage the TriNetX research network to systematically evaluate the incidence of tinnitus and all subtypes of hearing loss among individuals with documented COVID-19 infection, those who received COVID-19 vaccines (including mRNA and vector-based formulas), and matched unexposed comparator groups. Our aim is to clarify the risks of these outcomes, providing evidence to inform clinicians, researchers, and public health stakeholders regarding the auditory safety profiles of both infection and immunization in the ongoing management of the COVID-19 pandemic.

## Materials and methods

We conducted a retrospective cohort study utilizing the TriNetX Global Collaborative Network, a federated research platform that provides real-time access to de-identified electronic health records (EHRs) from healthcare organizations (HCOs) internationally. As of July 22, 2025, the Global Collaborative Network comprised 156 participating HCOs, encompassing 183,695,483 patient records. Adult patients (aged 18 years or older) were included and stratified into six distinct cohorts based on International Classification of Diseases, 10th Revision, Clinical Modification (ICD-10-CM) diagnosis codes, Current Procedural Terminology (CPT) codes, and Anatomical Therapeutic Chemical (ATC) codes: (1) individuals diagnosed with COVID-19; (2) recipients of an mRNA COVID-19 vaccine; (3) recipients of a viral vector-based COVID-19 vaccine; (4) individuals who never received an mRNA COVID-19 vaccine or diagnosis of COVID-19 infection (Comparator 2); (5) individuals who never received the viral vector vaccine or diagnosis of COVID-19 infection (Comparator 3); (6) individuals who never received any COVID-19 vaccine or diagnosis of COVID-19 infection (Comparator 1). Patients with a documented diagnosis of tinnitus (ICD-10-CM: H93.1) or conductive and sensorineural hearing loss (ICD-10-CM: H90) prior to the index event were excluded. The ICD-10-CM category H90 encompasses all subtypes of hearing loss, including conductive, sensorineural, mixed, and unspecified. Therefore, our analysis captured new-onset hearing loss of any type.

To ensure isolation of the exposure effect, cohorts were strictly defined to avoid cross-exposure. The COVID-19 infected cohort included only those with a documented diagnosis and excluded any individual who had received a COVID-19 vaccine at any time (prior to, during, or after the follow-up window). Similarly, the COVID-19 mRNA vaccinated and viral vector vaccinated cohorts included individuals who received the respective vaccines but excluded anyone with a documented COVID-19 diagnosis at any time during the study period or prior.

Vaccination status was determined using ATC codes for specific vaccine platforms. For the vaccine cohorts, inclusion required the completion of one dose of the specified vaccine. Individuals were excluded if they received a mixed series (e.g., an mRNA dose followed by a viral vector dose) to prevent confounding by vaccine platform. Comparator groups (1, 2, and 3) were strictly defined as individuals who had never received their respectively specified COVID-19 vaccine and had no documentation of COVID-19 infection at any point.

The index date of the COVID-19 infected cohort was defined as the date of the first documented diagnosis using the ICD-10-CM code U07.1 for COVID-19 infection. For the vaccination cohorts, the index date was defined as the date of the administration of the first vaccine dose. For all cohorts, the 6-month follow-up period for the primary outcomes began immediately following these respective index dates.

We utilized the ICD-10-CM category H90 (conductive and sensorineural hearing loss) as our primary metric for new-onset hearing loss. While this category serves as an aggregate for various subtypes, it was selected to provide a high-power, comprehensive assessment of all new-onset auditory pathology following exposure across the TriNetX network. We acknowledge that this aggregate approach captures a diverse range of pathologies, which were analyzed as a single clinical endpoint.

The six-month follow-up window was selected to ensure sufficient time for the clinical manifestation and diagnostic coding of post-exposure symptoms. This duration was chosen based on prior evidence of post-viral auditory sequelae and to provide a standardized interval that captures late-onset effects while maintaining the specificity of the association relative to the index event.

The primary study outcomes were the incidence of new diagnoses of tinnitus or conductive and sensorineural hearing loss within six months following the index event. Cohorts were established using ATC codes for vaccine types and ICD-10-CM codes for outcomes and exclusion criteria as appropriate. TriNetX’s analytic tools were employed to calculate odds ratios and other measures of association between comparison groups and outcomes. To reduce potential confounding, propensity score matching (PSM) was performed. For larger cohorts, patients were matched on age at index event, while smaller cohorts were matched on age, sex, race, and ethnicity.

To mitigate potential confounding related to healthcare-seeking behavior, we utilized PSM to balance cohorts on key demographic variables, which often serve as surrogates for healthcare utilization. However, we acknowledge as a limitation that our dataset did not allow for a direct adjustment based on the total number of healthcare encounters prior to the index date.

For cohorts exceeding 4 million patients, PSM was limited to age at index event due to technical constraints within the TriNetX platform that restrict multivariable matching for datasets of this magnitude. Age was prioritized as the primary matching variable given its role as the dominant confounder in auditory pathology. To account for this, cohorts with fewer than 4 million records were matched on age, sex, race, and ethnicity, to provide an internal validation of the associations across different levels of matching granularity.

TriNetX operates exclusively with de-identified data complying with the Health Insurance Portability and Accountability Act (HIPAA) Privacy Rule. Because this study involved only secondary analysis of de-identified aggregate data, institutional review board (IRB) approval was not required.

### Biostatistics

All analyses were conducted using the TriNetX analytics platform, which enables real-time creation and comparison of de-identified patient cohorts. The primary outcomes were new diagnoses of tinnitus (ICD-10-CM: H93.1) and conductive or sensorineural hearing loss (ICD-10-CM: H90) within six months of the index event (COVID-19 infection or vaccination). To minimize confounding, 1:1 propensity score matching (PSM) was performed; for larger cohorts (> 4 million patients) matching was based on age at index event, while smaller cohorts were matched on age, sex, race, and ethnicity. Adequacy of matching was confirmed using standardized mean differences (SMDs) with a threshold of < 0.1 to indicate good balance. Odds ratios (ORs) with 95% confidence intervals (CIs) were calculated using logistic regression to compare the likelihood of developing tinnitus or hearing loss between exposure and comparator cohorts, and absolute risk differences (RDs) with 95% CIs were reported to provide measures of clinical relevance. For very large cohorts with more than four million patients per group, the TriNetX platform permits propensity score matching on only a single variable and does not provide access to full patient level data for external analysis. As a result, detailed multivariable propensity score models, inverse probability weighting, and independent multivariable regression using exported individual records could not be performed at the scale of our primary cohorts. In this setting, age at index event was selected as the matching variable because it is the dominant risk factor for auditory outcomes. To approximate sensitivity analyses, we also constructed smaller sub-cohorts in which the platform allowed matching on multiple variables and performed 1:1 propensity score matching on age, sex, race, and ethnicity, then compared these effect estimates with those from the age matched primary cohorts. A p value of < 0.05 indicated a significant difference for all statistical tests.

## Results

We analyzed the incidence of newly diagnosed tinnitus and hearing loss within six months following COVID-19 infection or vaccination, reporting odds ratios (OR) and risk differences (RD) with 95% confidence intervals (CI) (Tables 1, 2, 3 and 4).

**Table 1:** Odds ratios for tinnitus within 6 months following COVID-19 infection or vaccination compared to various comparator groups. (* indicates statistical significance)

Odds Ratios for Tinnitus between COVID-19 Infected/Vaccinated/Comparator Groups			
Group 1 (Reference)	Group 2	Odds Ratio	95% CI
COVID-19 Infected	Comparator 1 (No mRNA or vector vaccines)	1.022	0.997–1.048
COVID-19 Infected	Comparator 2 (No mRNA vaccines)	1.001	0.977–1.025
COVID-19 Infected	Comparator 3 (No vector vaccines)	0.988	0.965–1.012
COVID-19 mRNA Vaccinated (No Infection History)	Comparator 2 (No mRNA vaccines)	*1.220	1.189–1.252
COVID-19 Vector Vaccinated (No Infection History)	Comparator 3 (No vector vaccines)	*1.269	1.114–1.447
COVID-19 Infected	COVID-19 mRNA Vaccinated (No Infection History)	*0.856	0.833–0.879
COVID-19 Infected	COVID-19 Vector Vaccinated (No Infection History)	0.972	0.866–1.092

**Table 2:** Odds ratios for hearing loss within 6 months following COVID-19 infection or vaccination compared to various comparator groups. (* indicates statistical significance)

Odds Ratios for Hearing Loss (All Subtypes) between COVID-19 Infected/Vaccinated/ Comparator Groups			
Group 1 (Reference)	Group 2	Odds Ratio	95% CI
COVID-19 Infected	Comparator 1 (No mRNA or vector vaccines)	*0.930	0.911–0.950
COVID-19 Infected	Comparator 2 (No mRNA vaccines)	*0.911	0.893–0.930
COVID-19 Infected	Comparator 3 (No vector vaccines)	*0.911	0.893–0.930
COVID-19 mRNA Vaccinated (No Infection History)	Comparator 2 (No mRNA vaccines)	*1.214	1.189–1.240
COVID-19 Vector Vaccinated (No Infection History)	Comparator 3 (No vector vaccines)	1.095	0.983–1.220
COVID-19 Infected	COVID-19 mRNA Vaccinated (No Infection History)	*1.313	1.285–1.343
COVID-19 Infected	COVID-19 Vector Vaccinated (No Infection History)	*0.873	0.786–0.969

**Table 3:** Risk differences for tinnitus within 6 months following COVID-19 infection or vaccination compared to various comparator groups after propensity score matching. (* indicates statistical significance)

Risk Differences for Tinnitus between COVID-19 Infected/Vaccinated/Comparator Groups								
Group 1	Group 2	Group 1 Sample Size	Group 2 Sample Size	Group 1 Risk	Group 2 Risk	Risk Difference	95% CI	*p*
COVID-19 Infected	Comparator 1 (No mRNA or vector vaccines)	2,077,146	2,083,227	0.603%	0.590%	0.013%	-0.002–0.028	0.0818
COVID-19 Infected	Comparator 2 (No mRNA vaccines)	2,230,588	2,241,071	0.607%	0.606%	0.001%	-0.014–0.015	0.9437
COVID-19 Infected	Comparator 3 (No vector vaccines)	2,187,374	2,197,312	0.602%	0.609%	-0.007%	-0.022–0.007	0.3408
COVID-19 mRNA Vaccinated (No Infection History)	Comparator 2 (No mRNA vaccines)	1,606,755	1,611,258	0.798%	0.655%	0.143%	0.124–0.162	*<0.0001
COVID-19 Vector Vaccinated (No Infection History)	Comparator 3 (No vector vaccines)	72,212	72,395	0.709%	0.559%	0.150%	0.068–0.231	*0.0003
COVID-19 Infected	COVID-19 mRNA Vaccinated (No Infection History)	1,489,828	1,493,066	0.679%	0.792%	-0.114%	-0.133 - -0.094	*<0.0001
COVID-19 Infected	COVID-19 Vector Vaccinated (No Infection History)	84,087	84,234	0.674%	0.693%	-0.019%	-0.098–0.060	0.6362

**Table 4:** Risk differences for hearing loss within 6 months following COVID-19 infection or vaccination compared to various comparator groups after propensity score matching. (* indicates statistical significance)

Risk Differences for Hearing Loss (All Subtypes) between COVID-19 Infected/Vaccinated/Comparator Groups								
Group 1	Group 2	Group 1 Sample Size	Group 2 Sample Size	Group 1 Risk	Group 2 Risk	Risk Difference	95% CI	*p*
COVID-19 Infected	Comparator 1 (No mRNA or vector vaccines)	2,068,889	2,078,200	0.823%	0.885%	-0.062%	-0.079 - -0.044	*<0.0001
COVID-19 Infected	Comparator 2 (No mRNA vaccines)	2,220,655	2,235,423	0.825%	0.904%	-0.080%	-0.097 - -0.062	*<0.0001
COVID-19 Infected	Comparator 3 (No vector vaccines)	2,220,981	2,235,525	0.824%	0.904%	-0.080%	-0.097 - -0.062	*<0.0001
COVID-19 mRNA Vaccinated (No Infection History)	Comparator 2 (No mRNA vaccines)	1,600,766	1,606,608	1.232%	1.017%	0.215%	0.192–0.238	*<0.0001
COVID-19 Vector Vaccinated (No Infection History)	Comparator 3 (No vector vaccines)	72,087	72,264	0.961%	0.879%	0.083%	-0.016–0.181	0.1002
COVID-19 Infected	COVID-19 mRNA Vaccinated (No Infection History)	1,487,108	1,483,105	1.233%	0.941%	0.292%	0.268–0.315	*<0.0001
COVID-19 Infected	COVID-19 Vector Vaccinated (No Infection History)	83,807	84,121	0.788%	0.901%	-0.114%	-0.201 - -0.026	*0.0110

### Tinnitus

Among adults with documented COVID-19 infection, the odds of developing tinnitus did not differ significantly from uninfected and unvaccinated controls (OR 1.022, 95% CI: 0.997–1.048; RD 0.013%, *p* = 0.0818). Similarly, no significant differences observed when comparing infected individuals with those unvaccinated for mRNA COVID-19 vaccines (OR 1.001: 95% CI 0.977–1.025; RD 0.001%, *p* = 0.9437) and viral vector COVID-19 vaccines (OR 0.988: 95% CI 0.965–1.012; RD -0.007%, *p* = 0.3408).

mRNA COVID-19 vaccination was associated with significantly higher odds of tinnitus compared to individuals with no history of infection or mRNA COVID-19 vaccination (OR 1.220: 95% CI 1.189–1.252; RD = 0.143%, *p* < 0.0001). Viral vector COVID-19 vaccination showed a similar association (OR 1.269: 95% CI 1.114–1.447; RD 0.150%, *p* = 0.0003).

When directly comparing COVID-19 infection to mRNA COVID-19 vaccination, infection was associated with lower odds of tinnitus than mRNA COVID-19 vaccination (OR 0.856: 95% CI 0.833–0.879; RD -0.114%, *p* < 0.0001), whereas no significant difference was found between infection and viral vector COVID-19 vaccination (OR 0.972: 95% CI 0.866–1.092; RD -0.019%, *p* = 0.6362).

### Hearing loss

An observed association was noted between COVID-19 infection and lower recorded odds of hearing loss diagnoses compared with individuals with no history of mRNA or viral vector COVID-19 vaccination (OR 0.930, 95% CI: 0.911–0.950; RD -0.062%, *p* < 0.0001). This association remained significant when comparing infected patients with mRNA-unvaccinated and viral vector unvaccinated comparators (OR 0.911, 95% CI: 0.893–0.93; RD -0.080%, *p* < 0.0001).

Conversely, mRNA COVID-19 vaccination was associated with higher odds of hearing loss compared with mRNA unvaccinated individuals (OR 1.214: 95% CI 1.189–1.240; RD 0.215%, *p* < 0.0001), while viral vector COVID-19 vaccination did not reach statistical significance (OR 1.095: 95% CI 0.983–1.220; RD 0.083, *p* = 0.1002).

Direct comparison shows higher odds of hearing loss following infection than after mRNA COVID-19 vaccination (OR 1.313: 95% CI 1.285–1.343; RD 0.219%, *p* < 0.0001) but lower odds compared to viral vector COVID-19 vaccination (OR 0.873: 95% CI 0.786–0.969; RD -0.114%, *p* = 0.0110).

### Sensitivity analyses

In smaller subcohorts where the TriNetX platform allowed matching on multiple characteristics, we repeated key exposure–comparator contrasts using 1:1 propensity score matching on age, sex, race, and ethnicity. For tinnitus, the odds ratio for mRNA vaccinated versus mRNA unvaccinated individuals in the primary age matched analysis was approximately 1.22 with a risk difference of about 0.14%, and in the multivariable matched sub-cohorts estimates remained in a similar range (odds ratios around 1.18–1.21 and risk differences around 0.12–0.15%), with overlapping confidence intervals. For hearing loss, the primary odds ratio of about 1.21 and risk difference of roughly 0.22% for mRNA vaccination were likewise mirrored by values in the range of roughly 1.18–1.20 and 0.20% in the more extensively matched sub-cohorts. These comparable estimates indicate that the direction and magnitude of the associations were stable across different matching specifications and support the robustness of the main findings despite the analytic constraints of the platform.

## Discussion

In this large, retrospective cohort study leveraging the TriNetX global health research network, we systematically evaluated the odds of new-onset tinnitus and hearing loss (all subtypes) following either COVID-19 infection or vaccination, juxtaposed against rigorously propensity-score-matched unvaccinated and uninfected comparators. Encompassing over 180 million patients, our analysis constitutes one of the largest real-world examinations to date of auditory sequelae stemming from the COVID-19 pandemic and its mitigation efforts. The results offer nuanced, clinically pertinent insights that challenge the prevailing assumptions and refine the risk-benefit narrative concerning postinfectious and post-vaccination auditory events.

In contrast to Dorney et al., our analysis was designed to assess the broader context of COVID-19 infection versus vaccination and to include hearing loss as an additional outcome. Differences in sample size compared with earlier TriNetX reports likely reflect our broader exclusion criteria and longer follow-up interval rather than a contraction of the underlying database.

Contrary to early studies that implicated SARS-CoV-2 as a direct etiologic agent for acute cochleovestibular disorders, our findings indicate that COVID-19 infection is not associated with a statistically significant increase in the odds of tinnitus relative to unvaccinated and uninfected comparators [10]. This result is consistent with emerging systematic reviews that have questioned the purported ototoxicity of SARS-CoV-2, particularly after accounting for background incidence rates and confounding variables [14, 20]. Interestingly, direct comparison between infected and vaccinated subgroups reveals a lower odds of tinnitus following infection than after mRNA or vector-based COVID-19 vaccination, despite the larger absolute number of patients exposed to the virus.

The observation that both mRNA and vector-based COVID-19 vaccines were associated with a modestly increased risk of new-onset tinnitus compared to their respective unvaccinated and uninfected comparators must be interpreted carefully. Although these risk elevations achieved statistical significance and persisted after propensity score matching, the risk increments were exceeding small. Given the very small absolute risk differences, these findings should be interpreted as signals detectable only in very large populations, rather than changes likely to be clinically apparent at the individual level. Nevertheless, these findings reinforce the importance of ongoing pharmacovigilance and suggest a potential, albeit rare, risk signal that warrants mechanistic investigation, particularly as vaccine platforms evolve.

Although the associations between vaccination and auditory symptoms were statistically significant, it is important to note that the absolute risk differences remained exceptionally low. These findings suggest that while a risk signal is detectable at the population level, the likelihood of a clinical event for an individual patient is minimal.

Findings for hearing loss were more complex. COVID-19 infection was associated with lower odds of hearing loss across all comparator groups, while mRNA COVID-19 vaccination showed the highest observed odds. The nonsignificant trend toward increased odds with viral vector COVID-19 vaccination may reflect limited sample size or residual confounding. These results may be influenced by heightened healthcare utilization and diagnostic coding in the period immediately following vaccination, as well as public awareness and reporting behavior. Increased public attention during vaccine rollout may have amplified reporting of coincidental symptoms, contributing to these findings.

The finding that COVID-19 infection was associated with lower recorded odds of hearing loss should not be interpreted as a biologically protective effect. Rather, this observed association is likely a result of detection and reporting bias. During the acute phase of COVID-19 infection, patients and providers naturally prioritize the management of life-threatening respiratory or systemic symptoms. This shift in clinical focus may lead to the under-diagnosis or delayed coding of secondary auditory symptoms compared to the uninfected control group or the vaccination cohorts, where patients may have been more sensitized to monitor or minor adverse events.

Several pathophysiological hypotheses could be posited to explain these findings. Immunologically mediated inner ear inflammation, transient microvascular alterations, or off-target activation of the innate or adaptive immune system have all been proposed as possible mechanisms underpinning vaccine-associated auditory complications, particularly in susceptible hosts [3, 21, 22]. While most proposed mechanisms relate to sensorineural involvement, conductive and mixed hearing loss may also arise through alternative pathways. SARS-CoV-2 has been isolated from the middle ear and mastoid, suggesting possible mucosal or Eustachian tube inflammation leading to transient conductive impairment [5]. Our retrospective study design precludes causal inference and cannot account for highly granular clinical variables, such as timing and severity of symptoms, audiometric phenotyping, or detailed medication and noise exposure histories. These limitations underscore the necessity of targeted prospective studies, including mechanistic and immunologic investigations, to validate and elucidate the nature of post-vaccination auditory symptoms.

The principal strength of this study lies in its unprecedented sample size, broad geographic and demographic representation, and rigorous design, including precise cohort definitions using ICD-10-CM, CPT, and ATC codes and robust propensity-score matching. These features enhance the generalizability and internal validity of our findings. Nonetheless, several limitations must be acknowledged. First, reliance on administrative codes may result in misclassification or underestimation of cases, particularly for transient or mild symptoms that do not require medical evaluation. Secondly, although propensity score matching was employed, comorbid conditions were not included in the matching process, leaving the possibility of residual confounding. Individuals with complex medical histories may be both more likely to receive vaccination and inherently at higher risk for auditory dysfunction. Thirdly, detailed clinical data such as symptom timing, audiometric confirmation, severity grading, and laterality were unavailable, limiting phenotypic characterization. In addition, we evaluated hearing loss using the aggregate ICD-10-CM H90 category rather than separating specific subtypes. This combined code includes conductive, sensorineural, mixed, and unspecified hearing loss and was chosen to capture all new-onset hearing loss diagnoses in a consistent way across institutions and exposure groups while preserving statistical power. A consequence of this approach is that we could not reliably examine subtype-specific patterns, which we acknowledge as a limitation and identify as an important focus for future studies with more detailed audiologic characterization. Our analysis also could not distinguish between first and subsequent vaccine doses. As such, our findings represent aggregate post-vaccination outcomes rather than dose-specific reactions. In addition, potential confounders including baseline hearing status, occupational or recreational noise exposure, and ototoxic medication use were not captured in the dataset. Finally, differences in health care-seeking behavior and increased surveillance during vaccination campaigns could have contributed to the observed associations with vaccination relative to infection.

Our analysis focused on the effects following the initial vaccine dose to isolate the risk associated with the primary immunological priming event. While this focus provides a clear index for capturing new-onset symptoms, a limitation of this approach is that we could not evaluate the cumulative risk or dose-specific effects associated with subsequent or booster doses.

It is imperative to consider the role of detection and surveillance bias in the interpretation of these findings. The period surrounding mass vaccination campaigns was characterized by unprecedented public awareness and active symptom monitoring. Consequently, individuals receiving a COVID-19 vaccine may have been more likely to seek medical evaluation for mild or transient auditory symptoms, such as tinnitus, that they might otherwise have ignored or managed at home. This surveillance effect could result in an artificial inflation of diagnostic coding within the vaccinated cohorts compared to the uninfected/unvaccinated control groups, who may not have been interacting with healthcare providers at the same frequency during the study window.

While we utilize a stratified PSM strategy, we acknowledge that a global multivariable adjustment or inverse probability of treatment weighting was not performed because, for cohorts of this size, the TriNetX environment restricts propensity score models to a single matching variable and does not permit export of full patient level data for more complex off-platform modeling. As a pragmatic alternative, we performed additional analyses in smaller sub-cohorts where matching on multiple demographic variables was feasible and found that the odds ratios and risk differences were very similar to those from the primary age matched analyses, supporting the robustness of our findings despite these analytic constraints.

Future prospective studies are warranted to validate these findings and clarify causal mechanisms. Standardized audiologic assessment protocols should be incorporated, including baseline and follow-up pure-tone audiometry, speech recognition testing, and tympanometry performed at defined intervals after vaccination or infection. Prospective collection of symptom onset timing, vaccine dose sequence, and detailed comorbid or medication history would further refine understanding of temporal and biological relationships.

## Conclusion

Our findings indicate that COVID-19 infection is not associated with increased odds of tinnitus or all types of hearing loss compared with uninfected and unvaccinated populations and appears to have lower recorded incidence than after mRNA COVID-19 vaccination, a pattern that is most plausibly explained by differences in diagnostic attention and surveillance rather than a true protective effect. Both mRNA and viral vector COVID-19 vaccines showed small but statistically significant increase in risk for new-onset tinnitus, and mRNA COVID-19 vaccines were also associated with hearing loss, although the absolute risk differences were very low. These results underscore the importance of patient counseling, transparent risk communication, and continued post-vaccination surveillance. While statistical associations were found in our large analysis, the clinical impact of these findings is tempered by the very small effect sizes observed and is unlikely to represent clinical significance. Prospective studies with standardized audiometric testing and mechanistic investigations are needed to confirm these associations and to guide otolaryngologic care, public health policy, and vaccine confidence.

## Data Availability

Data was obtained from the TriNetX database and is not publicly available due to data use restrictions.
